# A scalable multimodal framework for learning engagement recognition using three-dimensional convolutional neural networks and semi-automatic annotation

**DOI:** 10.3389/frai.2026.1798766

**Published:** 2026-05-08

**Authors:** Kuan-Cheng Lin, Chiung-Chen Tseng, Junyi Wu

**Affiliations:** Department of Management Information Systems, National Chung Hsing University, Taichung, Taiwan

**Keywords:** 3D CNN, behavior recognition, consistency analysis, facial emotion recognition, learning engagement recognition, multimodal learning analytics, semi-automatic annotation

## Abstract

This study developed a learning engagement recognition system that integrates behavioral and emotional modalities. By employing a three-dimensional (3D) convolutional neural network (CNN) model and a semi-automatic annotation mechanism, the developed system accurately analyzes students’ nonverbal behaviors and facially displayed emotions during class to identify their level of engagement. The study employed 570 instructional video recordings, segmented into 44,059 clips of 10s each. Three experts annotated the levels of emotional and behavioral engagement shown in these clips on the basis of standardized guidelines. Two separate 3D CNN models were trained to recognize emotional and behavioral features. Under the revised video-level validation setting, the emotional engagement model achieved an overall accuracy of 0.92, whereas the behavioral engagement model achieved an overall accuracy of 0.80. Moreover, Fleiss’ kappa was used to evaluate the consistency between model predictions and human annotations, indicating “almost perfect agreement.” The results demonstrate that integrating 3D convolutional neural networks with standardized, semi-automatic annotation rules can substantially enhance both the accuracy and scalability of automated learning engagement recognition systems while maintaining human-level reliability. The proposed framework provides a robust foundation for scalable AI-driven learning analytics and related video-based behavior recognition applications.

## 1 Introduction

Learning engagement is a critical factor influencing academic performance and learning investment among students (Pabba and Kumar, 2022; Ruiz et al., 2022). According to the framework proposed by Fredricks et al. (2004), learning engagement comprises behavioral, emotional, and cognitive dimensions. Behavioral engagement is shown through actions such as raising one’s hands and participating in discussions; emotional engagement is characterized by enthusiasm, interest, and curiosity; and cognitive engagement is defined by self-regulation and critical thinking. Previous studies have emphasized that behavioral and emotional engagement not only influence each other but also serve as key precursors to cognitive engagement (Li and Lerner, 2013; Liu et al., 2022; Pietarinen et al., 2014; Sathik and Jonathan, 2013).

In recent years, advances in artificial intelligence and computer vision have enabled the automatic sensing of learning engagement from observable behavioral and emotional signals, such as facial expressions, gaze direction, and body posture captured in classroom videos. From an AI system perspective, behavioral and emotional engagement are particularly important because they can be operationalized as observable, time-varying signals and serve as practical proxies for inferring students’ latent cognitive engagement. Consequently, the reliable and scalable recognition of behavioral and emotional engagement has become a foundational problem for AI-driven learning analytics and intelligent educational systems.

Facial expression recognition techniques have been widely adopted to detect emotional engagement levels because of their low cost and real-time responsiveness (Essa, 2002). In educational contexts, computer vision and machine learning have enabled the analysis of students’ facial expressions to help teachers monitor engagement levels and adjust their instruction accordingly (Abedi and Khan, 2021; Altuwairqi et al., 2021; Pabba and Kumar, 2022).

Behavioral engagement can also be identified through deep learning and object detection models. For instance, the Faster Region-Based Convolutional Neural Network model has been applied to detect classroom behaviors, such as raising hands or distracted behavior, thus improving the automation of interaction assessment (Khenkar and Jarraya, 2022; Pedro et al., 2013; Zheng et al., 2020).

Despite the aforementioned advances, two major challenges remain. First, engagement annotation standards have been inconsistent across studies, which have not established general operational definitions for emotional and behavioral features (Gupta et al., 2016; Whitehill et al., 2014). Second, most multimodal approaches focus on static feature analysis and fail to address the temporal dynamics of student engagement behavior (Mahmood et al., 2024; Miao et al., 2024).

From a system design perspective, effective learning engagement recognition requires not only high classification accuracy but also scalable annotation mechanisms and reliable alignment with human judgment. In real classroom settings, engagement recognition models must operate on temporally continuous video data and maintain consistency with expert annotation standards to be practically deployable. These requirements motivate the development of AI frameworks that jointly address temporal modeling, annotation efficiency, and human-level reliability.

To address the aforementioned limitations, this study developed (1) a multimodal learning engagement recognition system that integrates facial expressions and behavioral modalities and (2) a semi-automatic annotation process for enhancing labeling efficiency and consistency. This study makes three main contributions. First, it proposes consistent and practical guidelines for annotating emotional and behavioral engagement levels. Second, it develops modality-specific recognition models for learning behavior and learning emotion based on 3D CNNs. Third, it demonstrates the feasibility of recognizing human learning engagement through a semi-automatic annotation framework supported by deep learning.

## 2 Materials and methods

**Figure 1:**
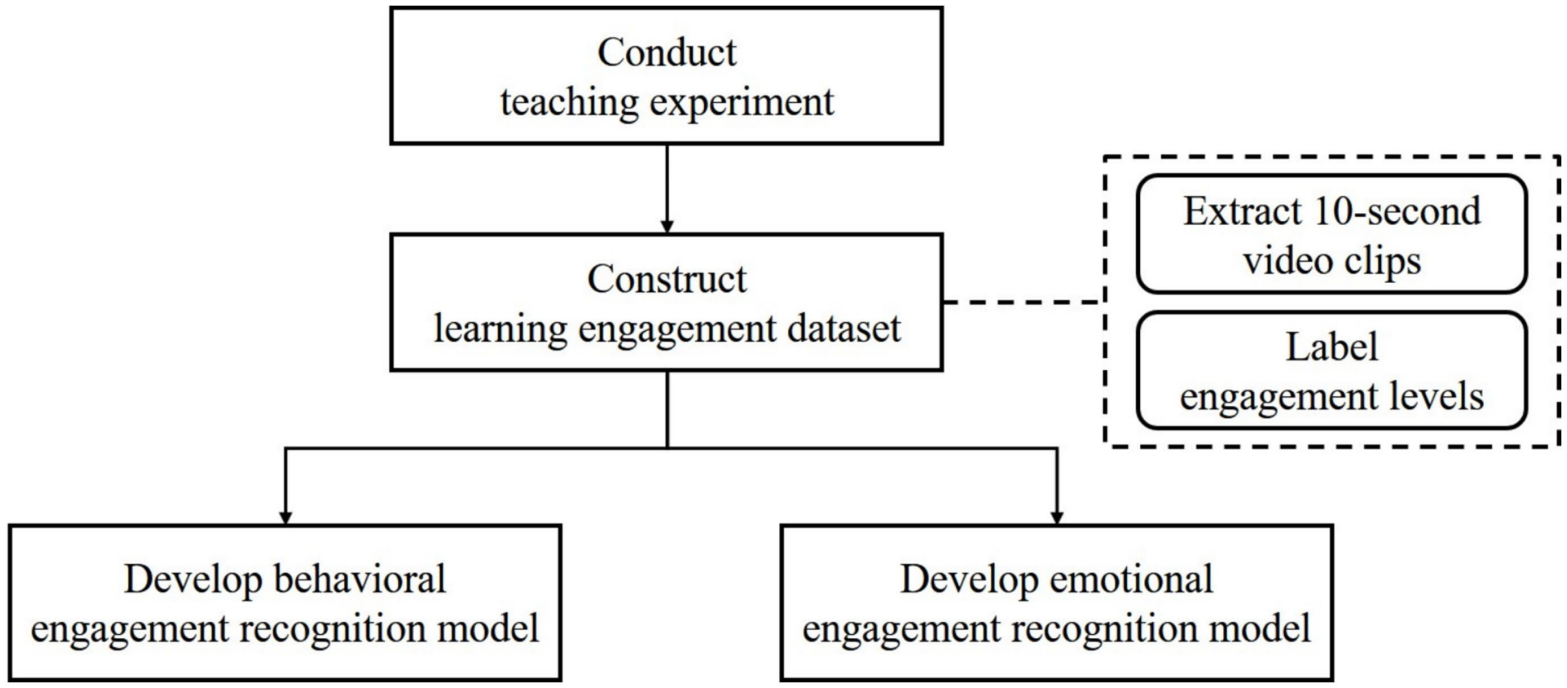
Research framework.

This study’s methodology comprised the following stages: instructional experiment conduct, dataset construction, data preprocessing, and construction of facial and behavioral engagement recognition models. The overall research process is illustrated in Figure 1.

### 2.1 Instructional experiment conduct

This study conducted a teaching experiment to collect data. In a computer laboratory, each student was seated in front of a computer equipped with a webcam so that video data could be recorded throughout the teaching process. Instructional content was broadcast to student terminals through presentation slides. The participants were 22 graduate students from the master’s program, all of whom provided informed consent prior to participation.

### 2.2 Dataset construction

This study constructed a learning engagement dataset and learning performance dataset. A total of 743 classroom videos were collected during the teaching experiment, with the durations of these videos ranging from 5 to 26 min. In accordance with the approach of Whitehill et al. (2014), the videos were segmented into 10s clips (approximately 300 frames per clip), resulting in a total of 44,059 video clips. These segments served as the fundamental units for subsequent analysis and model training. A 10-s temporal window was selected as a practical trade-off between capturing sufficient engagement dynamics and maintaining computational feasibility for 3D CNN–based temporal modeling.

Because multiple clips can be generated from the same raw classroom video, directly treating each clip as an independent sample during random partitioning may introduce a risk of data leakage. Therefore, in the revised experiment, this study adopted a video-level splitting strategy after clip generation, ensuring that clips derived from the same original video appeared in only one of the training, validation, or test sets. This design provided a stricter evaluation setting and reduced the risk of overlap across splits.

#### 2.2.1 Annotation process for learning engagement

To effectively annotate a large number of video segments, this study adopted a semi-automatic annotation process, combining manual and computer approaches to balance annotating efficiency and accuracy. The process consists of four main stages.

First, each 10-s video segment was assigned two labels, one indicating the level of emotional engagement and the other indicating the level of behavioral engagement. Labeling was performed manually through direct observation, and each segment was given a classification of either high engagement or low engagement on the basis of a weighted evaluation of predefined features. The annotation process was conducted collaboratively by three trained researchers, each with standardized training and at least 2 years of experience in emotion recognition research, to ensure high-quality annotations.

Next, using the aforementioned manually labeled data, the emotional and behavioral engagement recognition models were trained separately, and their predictive performance is monitored using a validation set. If the model accuracy has not yet reached a stable standard, training samples are added, parameters are adjusted, and retraining is performed.

Once the model achieves stable performance in the validation stage, the third stage begins, where the model is used for automatic labeling of large-scale segments, and validation is performed using manual sampling. Furthermore, this study uses Fleiss’s Kappa to measure the consistency between the model and human labeling, avoiding reliance solely on accuracy while ignoring the impact of random consistency. It also reflects whether the model successfully simulates the human labeling logic, thereby verifying the overall labeling quality. If the Fleiss’s Kappa value is higher than 0.8, further automatic labeling and expansion of the entire dataset can be performed; if the standard is not met, the labeling process is paused, high-information samples are added, and training is restarted.

Finally, through the above methods, we are able to continuously expand the database. This labeling strategy integrates manual labeling criteria with model prediction capabilities to establish a repeatable and consistent semi-automatic labeling process. This not only significantly improves the practical efficiency of processing large amounts of data, but also ensures the quality, reliability, and scalability of the data required for subsequent deep learning model training.

#### 2.2.2 Pilot study of the annotation process

To minimize subjectivity in the design of annotation criteria and feature weighting, a pilot study was conducted prior to the formal annotation of emotional and behavioral engagement. Thirty 10-s video segments were randomly selected from the classroom recordings for frame-by-frame manual observation and labeling.

For emotional engagement, the annotations focused on gaze direction and mouth activity, whose correlations with overall perceived emotional engagement were assessed. Pearson correlation analysis was then conducted to assess the relationships between these features and overall perceived emotional engagement. The results indicated that perceived emotional engagement was more strongly correlated with gaze direction (Pearson’s *r* = 0.79) than with mouth activity (*r* = 0.48). Accordingly, the weights of gaze direction and mouth activity were set to 0.6 and 0.4, respectively.

For behavioral engagement, three observable features were examined: head orientation, hand movement, and body posture. Their correlations with overall engagement rating were 0.52, 0.39, and 0.39, respectively; thus, their feature weights were set to 0.4, 0.3, and 0.3, respectively.

The researchers observed that when students maintained their focus on the screen or sat with an upright posture for more than 8 s within a clip, they were consistently rated as being highly engaged; conversely, students in segments showing less sustained behavior were rated as having low engagement. This temporal criterion was unanimously adopted by the annotators and subsequently applied throughout the entire annotation process.

#### 2.2.3 Emotional engagement dataset

An emotional engagement dataset was designed to assess the students’ emotional engagement during the learning process on the basis of their facial features. The primary observational indicators were gaze direction and mouth activity. Annotators assigned an engagement score to each segment on the basis of the observed features, with scores of 1 and 2 indicating low and high engagement, respectively. The detailed annotation criteria are presented in Table 1.

**Table 1:** Criteria for annotating emotional engagement.

Engagement level	Eye gaze criteria (weight: 0.6)	Mouth activity criteria (weight: 0.4)
Low engagement	Gaze directed forward for ≤8 s (e.g., eyes closed and looking away)	Noticeable mouth activity (e.g., talking, chewing, and yawning)
High engagement	Eyes steadily directed forward; gaze remains fixed	Mouth naturally closed; no significant movement

On the basis of the aforementioned criteria, manual annotation was conducted to assign engagement scores for both eye gaze and mouth activity. The overall emotional engagement score for each video segment was then calculated using the following weighted formula:

$$\begin{array}{l} \text{Emotional engagement score}=(\mathrm{Eye}\;\text{score}\times 0.6)\\ +(\text{Mouth score}\times 0.4) \end{array}$$

Finally, the calculated score was used to assign a label of high engagement or low engagement to each segment in accordance with the classification criteria outlined in Table 2.

**Table 2:** Classification of emotional engagement level.

Engagement level	Score range
Low	1.0–1.4
High	1.5–2.0

For example, if a student in a video segment did not maintain eye contact with the screen for most of the time (eye score = 1) and showed no noticeable mouth activity (mouth score = 2), the emotional engagement score for that segment was calculated as (1 × 0.6) + (2 × 0.4) = 1.4.

#### 2.2.4 Behavioral engagement dataset

The constructed behavioral engagement dataset comprised images of the students’ observable behaviors in a classroom. The features in this dataset were head posture, hand movement, and body posture, which are indicators of behavioral engagement. Annotators assigned a score of 1 (low) or 2 (high) to each behavioral feature on the basis of direct observation. The detailed annotation criteria for behavioral engagement are provided in Table 3.

**Table 3:** Criteria for annotating behavioral engagement.

Engagement level	Head posture (weight: 0.4)	Hand movement (weight: 0.3)	Body posture (weight: 0.3)
Low engagement	Facing the screen for ≤8 s (e.g., turning head and looking down)	Irrelevant hand activity (e.g., using a phone)	Facing the screen for ≤8 s (e.g., lying on desk and leaning sideways)
High engagement	Head orientation, hand gestures, and body posture do not indicate distracted behavior.		

Behavioral engagement scores were calculated using the following weighted equation:

$$\begin{array}{l} \text{Behavioral engagement score}=(\text{Head posture score}\times 0.4)\\ +(\text{Hand movement score}\times 0.3)+(\text{Body posture score}\times 0.3) \end{array}$$

On the basis of the computed scores, each segment was classified as representing high engagement or low engagement according to the criteria defined in Table 4.

**Table 4:** Classification of behavior engagement level.

Engagement level	Score range
Low	1.0–1.6
High	1.7–2.0

This well-defined and systematic annotation procedure ensured that the constructed behavioral engagement dataset accurately reflected the students’ actual engagement during instruction. Therefore, this dataset can serve as a reliable foundation for training future behavior recognition models.

### 2.3 Data preprocessing

To enhance the model’s ability to recognize emotional and behavioral engagement features, a systematic data preprocessing procedure was applied prior to training. The raw video data were transformed into structured image formats, with separate strategies designed for emotional engagement recognition and behavioral engagement recognition.

Because student behavior unfolds through motion over time, such behavior cannot be captured by single-frame images alone. Therefore, this study used 10-s video segments (300 frames at 30 fps) as the basic unit of analysis, allowing the model to retain and learn from the temporal variations in facial expressions and postures.

#### 2.3.1 Emotional engagement recognition

Emotional engagement recognition centered on the students’ facial expressions, gaze direction, and mouth movements, which represent subtle microexpressions that are sensitive to variations in lighting, occlusion, and pose. To preserve these fine-grained details, high-resolution and color-rich images were required to enhance recognition performance.

This study used OpenFace (Baltrušaitis et al., 2016) to extract 68 facial landmarks, including key regions such as the eyes, mouth, eyebrows, and nose. From these points, features such as eye openness, mouth movement, and head orientation were derived, all of which provide meaningful emotional cues. To retain color and lighting information, input images were kept in the RGB format. Each clip was represented as 300 consecutive facial images with a size of 32 × 32 × 3; these images were input into the model, allowing the model to learn how emotion is dynamically expressed over time.

#### 2.3.2 Behavioral engagement recognition

Behavioral engagement recognition centered on the students’ overall body movements and postures, such as whether they were facing the screen, turning their heads, using unrelated hand gestures (e.g., mobile phone use), or slouching. These actions typically involve large spatial variations and rely more on posture and silhouette than on texture or color details. Therefore, input images were converted to grayscale (32 × 32 × 1) to reduce dimensionality and computational load while preserving the structural features of the head, hands, and body. These grayscale image sequences were then used to train the model, improving efficiency without sacrificing essential motion-related information.

### 2.4 Learning engagement recognition models

To effectively identify students’ learning engagement in classroom settings, this study developed separate recognition models for emotional engagement and behavioral engagement. Given that the input data comprised temporally continuous video segments (each lasting 10 s and containing 300 frames), a three-dimensional (3D) convolutional neural network (CNN) was selected as the primary model architecture. Separate modality-specific models were adopted to allow each 3D CNN to specialize in capturing modality-dependent temporal patterns, avoiding potential interference introduced by early feature fusion.

#### 2.4.1 Emotional engagement recognition model

The developed emotional engagement recognition model uses RGB facial images (300 in this study) extracted from each video segment as its input to capture subtle changes in students’ facial expressions, such as their gaze direction and mouth activity, during classroom sessions. RGB images were retained to preserve fine-grained color and texture cues that are informative for facial expression analysis and gaze-related emotional engagement recognition.

All images were uniformly scaled to a resolution of 32 × 32 × 3 and trained using labeled data. The model architecture employs a combination of multi-layer 3D convolutions and pooling to capture the changing trends of facial features over time, further predicting the emotional engagement (high/low) of each video segment. This model is for a binary classification task, using a sigmoid output function and binary crossentropy as the loss function. Dropout regularization was incorporated during training to prevent overfitting, and the Adam optimizer was used for parameter updates.

The hyperparameters of the facial engagement recognition model are shown in Table 5.

**Table 5:** Hyperparameter settings for the emotional engagement recognition model.

Hyperparameter	Value
Batch size	4
Epochs	50
Optimizer	Adam
Learning rate	0.001
Loss function	Binary cross entropy
Activation	ReLU, sigmoid (output layer)
Regularization parameters	Dropout (0.5), L2 regularization (0.01)

#### 2.4.2 Behavioral engagement recognition model

The behavioral engagement recognition model focuses on students’ overall body movements and postures, such as whether they are looking directly at the screen or exhibiting distracting behaviors (e.g., turning their head, lying down, fidgeting with objects), to determine whether students are focused and engaged. In addition, grayscale images were used to emphasize structural posture and motion patterns while reducing input dimensionality and computational cost.

This model consists of 3D convolution and pooling layers, and its output layer includes a sigmoid activation function for performing binary classification. The model uses the binary cross-entropy loss function to measure prediction error. During training, dropout regularization is used to prevent overfitting, and the Adam optimizer is employed for parameter updates.

The hyperparameter settings of the behavioral engagement recognition model are shown in Table 6.

**Table 6:** Hyperparameter settings for the behavioral engagement recognition model.

Hyperparameter	Value
Batch size	8
Epochs	50
Optimizer	Adam
Learning rate	0.001
Loss function	Binary cross entropy
Activation	ReLU, Softmax (output layer)
Regularization parameters	Dropout (0.5)

## 3 Results

### 3.1 Learning engagement recognition models

To effectively capture the participants’ in-class engagement states, this study developed two recognition models: one for emotional engagement and another for behavioral engagement. These two models were trained on facial features and behavioral cues, respectively. The following sections present results from evaluations of both models across different training stages.

#### 3.1.1 Prediction of emotional engagement

The emotional engagement recognition model was trained in stages by using manually annotated data. This approach was used to observe how prediction performance varied with dataset size. The training began with 500 samples and was gradually expanded to 4,000 samples. At each stage, the model’s accuracy on the validation set was evaluated.

The results clearly indicated that accuracy increased with the training set size, peaking at 94% when the training set contained 3,500 samples. The detailed training results obtained for the emotional engagement recognition model are presented in Table 7.

**Table 7:** Training performance of the emotional engagement recognition model.

Number of training set samples	Accuracy
500	84%
1,500	85%
2,500	91%
3,500	94%
4,000	90%

On the basis of the aforementioned results, 3,500 labeled samples were used to train the emotional engagement recognition model, which was then used in large-scale automatic annotation and prediction tasks.

This model not only demonstrated strong classification accuracy but also exhibited stable generalization performance. The model successfully identified 21 high-participation samples and 38 low-participation samples, with only 5 samples misclassified, achieving an overall accuracy of 92%. Further metrics showed that both precision and recall were shown in Table 8. The scores indicates the model’s consistent and reliable classification between high and low engagement levels but also balances accuracy and sensitivity, demonstrating high application potential. In other words, the observed performance saturation suggests that the 3D CNN effectively captured the dominant temporal patterns of emotional engagement within the available data scale.

**Table 8:** Metrics of the emotional engagement recognition model on the validation dataset.

	Precision	Recall	F1-score	Support
Class 0	0.93	0.95	0.94	40
Class 1	0.91	0.88	0.89	24
Accuracy			0.92	64
Macro avg	0.92	0.91	0.92	64
Weighted avg	0.92	0.92	0.92	64

#### 3.1.2 Behavioral engagement prediction

The behavioral engagement recognition model was also trained under a stepwise increase in the number of manually labeled data samples. This approach was adopted to examine how model performance varied with dataset size. The aforementioned model used 300 grayscale images extracted from each 10-s video segment as its input, focusing on capturing students’ overall bodily movements and postural features, such as head turning, body leaning, and hand activity, all of which are observable behaviors.

The accuracy of the behavioral engagement recognition model steadily increased with the size of the training set, with the model achieving its highest accuracy of 93% when trained on 2,000 samples. Larger sample sizes did not yield further improvement and even led to a slight decline in accuracy, suggesting that the model had converged and that additional data had limited influence on learning performance. The training results of the behavioral engagement recognition model are summarized in Table 9.

**Table 9:** Training performance of the behavioral engagement recognition model.

Number of training set samples	Accuracy
500	89%
1,000	90%
2,000	93%
2,500	90%

On the basis of the aforementioned results, the behavioral engagement recognition model was trained using 2,000 labeled samples and then evaluated on the validation dataset, exhibiting a strong balance between accuracy and computational efficiency.

Table 10 displays the confusion matrix of the aforementioned model on the validation dataset, showing that the model has good classification stability and identification efficiency. The model successfully identified 45 high-participation samples and 35 low-participation samples, with 20 misclassifications in each category. The overall accuracy reached 80%, with the F1-score at 81%, indicating that the model can not only accurately determine whether students participate in class, but also is unlikely to miss actual high participants. This shows that the model has high practicality and portability, and is sufficient to be applied to subsequent large-scale behavioral data automatic identification and dynamic monitoring tasks.

**Table 10:** Metrics of the behavioral engagement recognition model on the validation dataset.

	Precision	Recall	F1-score	Support
Class 0	0.70	0.88	0.78	40
Class 1	0.90	0.75	0.82	60
Accuracy			0.80	100
Macro avg	0.80	0.81	0.80	100
Weighted avg	0.82	0.80	0.80	100

### 3.2 Validation of annotation consistency and reliability

The consistency and reliability of the learning engagement annotation models were tested by comparing their predictions against manual annotations. The best-performing models for emotional engagement and behavioral engagement were then selected for consistency evaluation. The correspondence between automated annotations and human labels was assessed for each modality.

In the staged training process, the emotional and behavioral engagement recognition models achieved peak accuracy values of 94 and 93%, respectively. Under the revised video-level validation setting, the final emotional and behavioral models achieved overall accuracies of 0.92 and 0.80, respectively. However, accuracy (reflecting the overall proportion of correct predictions) alone does not fully indicate whether a model has learned to emulate the logic underlying human annotation. Therefore, Fleiss’ kappa coefficient was used to evaluate the degree of agreement between model predictions and manual labels (Fleiss, 1971). This coefficient accounts for chance agreement in the annotation results, making it a more comprehensive metric for evaluating whether a model has the ability to simulate the outputs of human annotators. According to the guidelines proposed by Landis and Koch (1977), a kappa value between 0.81 and 1.00 indicates “almost perfect agreement,” suggesting that the model closely replicates skilled human judgment.

**Figure 2:**
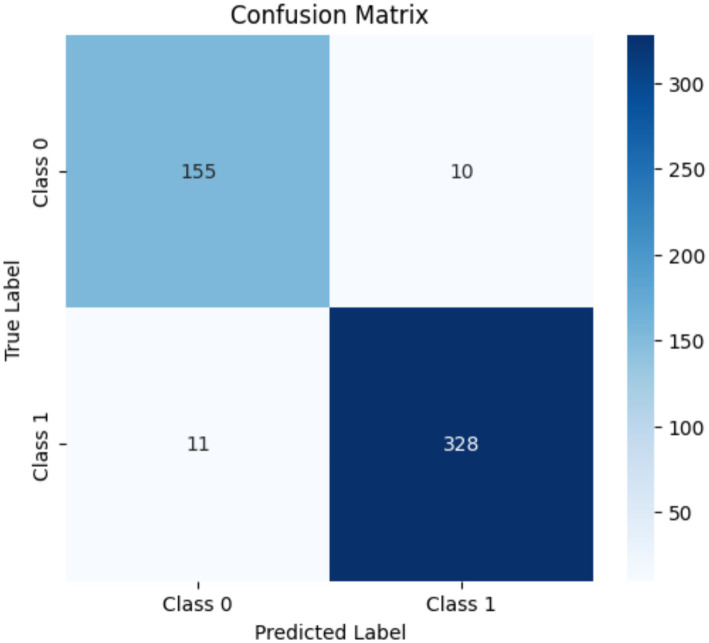
Confusion matrix for consistency in emotional engagement annotation (manual annotations vs. model predictions).

**Figure 3:**
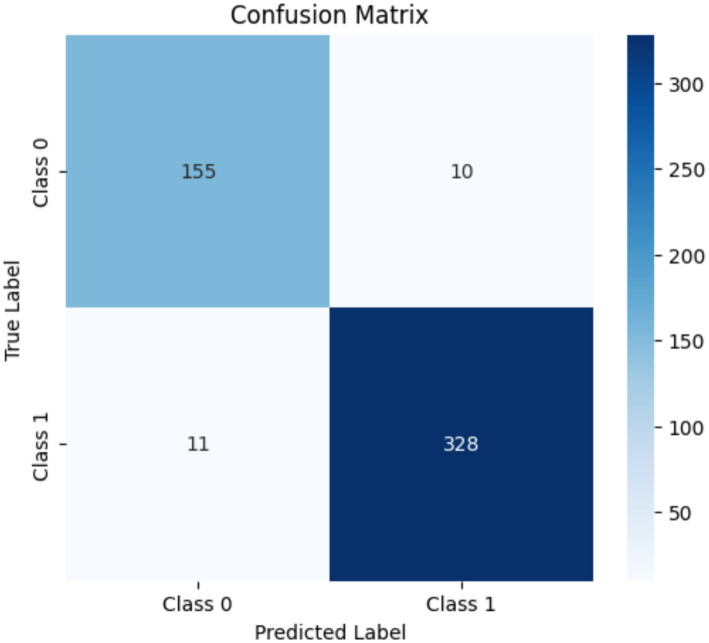
Confusion matrix for consistency in behavioral engagement annotation (manual annotations vs. model predictions).

The emotional and behavioral engagement recognition models achieved kappa values of 0.91 (Figure 2) and 0.94 (Figure 3), respectively. Both values fall within the “almost perfect agreement” range, demonstrating that the models show high reliability and practical utility in the recognition of students’ emotional states and observable behaviors.

These results confirm the effectiveness and reliability of the semiautomated annotation strategy proposed in this study and provide a sound foundation for large-scale annotation and future model deployment.

In summary, the semiautomated annotation process and deep learning models proposed in this study substantially enhance annotation efficiency while maintaining strong alignment with skilled human judgment in terms of accuracy and consistency. These results indicate that the proposed models not only achieve high classification accuracy but also replicate expert annotation logic, which is critical for reliable semi-automatic labeling and downstream learning analytics.

## 4 Discussion

This study developed an integrated learning engagement recognition model that combines facial expressions and behavioral modalities. Moreover, a semi-automatic annotation process was designed to construct a high-consistency training dataset for 3D CNN models capable of capturing temporal dynamics. Compared with previous studies, this work introduces notable innovations in methodology, model design, and practical applications. The following discussion is organized around four key aspects of this study’s contributions: model innovation, annotation strategy, modality-specific design, and application potential.

### 4.1 Methodological characteristics and comparison with previous studies

In contrast to traditional approaches that rely on static image-based features or frame-level aggregation, this study adopts a temporal modeling strategy that involves inputting full 10-s (300-frame) video clips to 3D CNN models. This strategy enables the models to capture dynamic changes in student engagement over time, which are often overlooked in static representations.

The adoption of modality-specific models rather than early fusion of features allows each 3D CNN model to specialize in capturing the subtle patterns of facial expressions and body movements. Furthermore, the proposed annotation strategy not only improves labeling efficiency but also ensures consistency through empirical feature weighting and inter-rater reliability validation.

### 4.2 Practical validation of the semi-automatic annotation process

This study employed a semi-automatic labeling process to assist human annotators in annotating 44,059 video segments with emotional and behavioral engagement scores. On average, this approach substantially saved the manual annotation time. The consistency between system-generated and human annotations was evaluated using Fleiss’ kappa, yielding high agreement values of 0.91 for emotional engagement and 0.94 for behavioral engagement. These results confirm the feasibility of applying the proposed approach for the construction of large-scale engagement datasets with reliable consistency.

### 4.3 Analysis of modality-specific recognition features

The constructed emotion recognition model focuses on fine-grained facial cues such as gaze direction and mouth activity, which are effective indicators of internal learning motivation and emotional states. By contrast, the constructed behavioral recognition model captures gross motor cues, including body orientation and hand movements, which are particularly valuable in cases where facial cues are unavailable or ambiguous (e.g., occlusion). By training separate models for each modality, the proposed system achieves greater flexibility and extensibility, allowing it to adapt to diverse classroom conditions and student behaviors.

From an AI system perspective, the results highlight the importance of jointly considering temporal modeling, annotation efficiency, and human–AI agreement in engagement recognition tasks. High classification accuracy alone is insufficient for practical deployment if model predictions are misaligned with expert judgment. The proposed framework demonstrates that semi-automatic annotation combined with modality-specific temporal modeling can achieve promising and practically usable performance while maintaining scalability, which is essential for real-world video-based learning analytics systems.

### 4.4 Practical application potential and future research directions

From an AI system deployment perspective, the proposed framework has considerable potential for real-world applications. It can serve as a core module for real-time engagement monitoring in smart classrooms, enabling intelligent systems to support instructors by providing immediate insights into students’ attentional states and facilitating adaptive instructional pacing. Moreover, both the semi-automatic annotation process and the 3D CNN–based modeling approach are transferable to other video-based behavior analytics tasks, such as attention tracking or anomaly detection. Future research may incorporate additional modalities and investigate deployment across diverse instructional environments and video conditions, thereby further advancing scalable and reliable AI-driven learning analytics systems.

## 5 Conclusion

This study developed a multimodal learning engagement recognition framework that integrates facial and behavioral modalities using 3D convolutional neural networks and a semi-automatic annotation strategy. By jointly addressing temporal modeling, annotation efficiency, and human–AI consistency, the proposed framework demonstrates that reliable learning engagement recognition can be achieved at scale while maintaining alignment with expert judgment. These findings establish a robust foundation for downstream engagement analysis and future AI-driven learning analytics applications.

Several limitations of this study suggest directions for future research. First, the dataset was collected from graduate-level courses in information management, resulting in relatively homogeneous learning contexts. Future studies should evaluate the proposed framework across diverse educational levels, disciplines, and instructional settings to further assess its generalizability. Second, the current framework focuses on emotional and behavioral engagement modalities. Additional signals, such as speech, eye movement, or interaction traces, may be incorporated to support more comprehensive multimodal engagement modeling.

Finally, the proposed models were evaluated in an offline setting. Future work may explore system-level integration with real-time processing pipelines and deployment-oriented architectures to support practical implementation in real classroom environments.

## Data Availability

The raw data supporting the conclusions of this article will be made available by the authors, without undue reservation.

## References

[ref1] Abedi A. Khan S.S. (2021) "Improving state-of-the-art in detecting student engagement with resnet and tcn hybrid network," in 18th Conference on Robots and Vision (CRV), (Burnaby, BC: IEEE). 151–157.

[ref2] Altuwairqi K. Jarraya S. K. Allinjawi A. Hammami M. (2021). A new emotion–based affective model to detect student’s engagement. J. King Saud Univ.-Comput. Inform. Sci. 33, 99–109. doi: 10.1016/j.jksuci.2018.12.008

[ref3] Baltrušaitis T. Robinson P. Morency L. P. (2016). “OpenFace: an open source facial behavior analysis toolkit,” in 2016 IEEE Winter Conference on Applications of Computer Vision (WACV), (Lake Placid, NY: IEEE), 1–10.

[ref4] Essa I. A. (2002). Ubiquitous sensing for smart and aware environments. IEEE Pers. Commun. 7, 47–49. doi: 10.1109/98.878538

[ref5] Fleiss J. L. (1971). Measuring nominal scale agreement among many raters. Psychol. Bull. 76, 378–382. doi: 10.1037/h0031619

[ref6] Fredricks J. A. Blumenfeld P. C. Paris A. H. (2004). School engagement: potential of the concept, state of the evidence. Rev. Educ. Res. 74, 59–109. doi: 10.3102/00346543074001059

[ref7] Gupta A. D'Cunha A. Awasthi K. Balasubramanian V. (2016). Daisee: Towards user Engagement Recognition in the wild. arXiv preprint. Available online at: https://arxiv.org/abs/1609.01885 (Accessed January 28, 2026).

[ref8] Khenkar S. Jarraya S. K. (2022). Engagement detection based on analyzing micro body gestures using 3D CNN. Comput. Mater. Continua 70, 2655–2677. doi: 10.32604/cmc.2022.019152

[ref9] Landis J. R. Koch G. G. (1977). The measurement of observer agreement for categorical data. Biometrics 33, 159–174. doi: 10.2307/2529310 843571

[ref10] Li Y. Lerner R. M. (2013). Interrelations of behavioral, emotional, and cognitive school engagement in high school students. J. Youth Adolesc. 42, 20–32. doi: 10.1007/s10964-012-9857-5, 23180069

[ref11] Liu S. Liu S. Liu Z. Peng X. Yang Z. (2022). Automated detection of emotional and cognitive engagement in MOOC discussions to predict learning achievement. Comput. Educ. 181:104461. doi: 10.1016/j.compedu.2022.104461

[ref12] Mahmood N. Bhatti S. M. Dawood H. Pradhan M. R. Ahmad H. (2024). Measuring student engagement through behavioral and emotional features using deep-learning models. Algorithms 17:458. doi: 10.3390/a17100458

[ref13] Miao Q. Li L. Wu D. (2024). Correction: an English video teaching classroom attention evaluation model incorporating multimodal information. J. Ambient. Intell. Humaniz. Comput. 15, 3715–3715. doi: 10.1007/s12652-024-04845-4

[ref14] Pabba C. Kumar P. (2022). An intelligent system for monitoring students' engagement in large classroom teaching through facial expression recognition. Expert. Syst. 39:e12839. doi: 10.1111/exsy.12839

[ref15] Pedro M. O. Baker R. Bowers A. Heffernan N. (2013). Predicting College Enrollment from Student Interaction with an Intelligent Tutoring system in Middle School. Memphis, Tennessee: Educational Data Mining.

[ref16] Pietarinen J. Soini T. Pyhältö K. (2014). Students’ emotional and cognitive engagement as the determinants of well-being and achievement in school. Int. J. Educ. Res. 67, 40–51. doi: 10.1016/j.ijer.2014.05.001

[ref17] Ruiz N. Yu H. Allessio D. A. Jalal M. Joshi A. Murray T. . (2022). ATL-BP: a student engagement dataset and model for affect transfer learning for behavior prediction. IEEE Trans. Biomet. Behav. Identity Sci. 5, 411–424. doi: 10.1109/TBIOM.2022.3210479

[ref18] Sathik M. Jonathan S. G. (2013). Effect of facial expressions on student’s comprehension recognition in virtual educational environments. Springerplus 2:455. doi: 10.1186/2193-1801-2-455, 24130957 PMC3795200

[ref19] Whitehill J. Serpell Z. Lin Y. C. Foster A. Movellan J. R. (2014). The faces of engagement: automatic recognition of student engagementfrom facial expressions. IEEE Trans. Affect. Comput. 5, 86–98. doi: 10.1109/TAFFC.2014.2316163

[ref20] Zheng R. Jiang F. Shen R. (2020) "Intelligent student behavior analysis system for real classrooms," in: ICASSP 2020–2020 IEEE International Conference on Acoustics, Speech and Signal Processing (ICASSP), (Barcelona: IEEE). p. 9244–9248.

